# The Varying Histology of Hepatic Sarcoidosis and the Relation of Bile Duct Damage and Loss to the Presence of Portal Hypertension and Cirrhosis

**DOI:** 10.1016/j.gastha.2024.10.001

**Published:** 2024-10-10

**Authors:** Divya B. Dasani, Maria Isabel Fiel, Camila C. Simoes, Adam S. Morgenthau, Thomas D. Schiano

**Affiliations:** 1 Recanati-Miller Transplantation Institute at Icahn School of Medicine at Mount Sinai, New York, New York; 2 Department of Pathology, Molecular and Cell-Based Medicine Icahn School of Medicine at Mount Sinai, New York, New York; 3 Department of Medicine and Division of Pulmonary, Critical Care and Sleep Medicine, Department of Pulmonology, Icahn School of Medicine at Mount Sinai, New York, New York; 4 Department of Pathology and Division of Gastrointestinal, Hepatobiliary, and Pancreas at the University of Arkansas for Medical Sciences, Little Rock, Arkansas

**Keywords:** Liver biopsy, Granulomas, Cholestasis, Biliary cirrhosis, Bile duct loss

## Abstract

**Background and Aims:**

Sarcoidosis is a multisystem disorder characterized by nonnecrotizing granulomas. Studies suggest 20%–70% of patients with sarcoidosis have abnormal liver chemistries or abdominal imaging. Hepatic sarcoidosis may be complicated by portal hypertension (portal HTN) with or without cirrhosis. Few studies have reviewed the liver histopathology of sarcoidosis.

**Methods:**

Searching the pathology database using the terms “sarcoidosis” and “liver,” patients were identified and cross-referenced to patients in the Sarcoidosis Clinic. Patients met the diagnostic criteria for sarcoidosis. Those with isolated granulomatous hepatitis were excluded. Demographics, abdominal imaging, biochemistries, and detailed histological features were cataloged.

**Results:**

Patients were separated into 2 groups: those with portal HTN with or without cirrhosis (pHTN+) and those without portal HTN (pHTN-). Fifty-three patients had biopsies available for review (pHTN+, n = 33; pHTN-, n = 20). The groups did not differ in the location, type, or number of granulomas. The pHTN + group had more bile duct damage (*P* = .025) and loss (*P* = .019). Patients in the pHTN + group also had biliary cirrhosis, nodular regenerative hyperplasia, or outflow obstruction.

**Conclusion:**

There are several causes for portal HTN in sarcoidosis. Thus, liver biopsy is essential in its evaluation. Bile duct damage and loss are associated with the presence of portal HTN and cirrhosis. Biliary abnormalities may occur independently of granulomatous inflammation, and can thus identify a subset of patients at risk for progressive liver disease.

## Introduction

Sarcoidosis is a multisystem disease that is commonly characterized by nonnecrotizing epithelioid granulomas. It can involve any organ, most commonly the lungs.^1^ Clinical presentations vary depending on the organ affected; many patients have subclinical disease. Hepatic involvement in sarcoidosis is often subclinical or discovered incidentally. In the U.S., patients with hepatic sarcoidosis are most commonly middle-aged, African American, and female.^2^ Studies demonstrate varying rates (6%–80%) of sarcoidosis involving the liver.^3^ Symptomatic patients often present with abdominal pain, pruritus, hepatosplenomegaly, fever, night sweats, or weight loss.^1^ In more advanced cases, patients may develop portal hypertension (portal HTN) and liver failure.

Currently, clinicians diagnose sarcoidosis utilizing guidelines established by the American Thoracic Society (ATS), which provides recommendations for the utility of certain diagnostic tests when evaluating patients with suspected or known sarcoidosis. The diagnosis of sarcoidosis is based on 3 criteria: 1. compelling clinical presentation, 2. presence of nonnecrotizing granulomatous inflammation in one or more tissue samples, and 3. excluding alternate granulomatous diseases.^4^ For organs like the liver, there are few criteria to diagnose hepatic sarcoidosis precisely. Clinical markers that support the diagnosis of hepatic sarcoidosis are elevated liver chemistry tests and radiographic imaging abnormalities demonstrating hepatomegaly and hepatic nodules with or without abdominal lymphadenopathy, especially periportal adenopathy.^4^ Clinicians may observe increases in alkaline phosphatase (ALP) and gamma-glutamyl transferase (GGT), which are most commonly elevated in hepatic sarcoidosis.^5^, ^6^, ^7^, ^8^ According to ATS, of the 12% of patients with liver chemistry abnormalities, most will have hepatic granulomas if biopsied.

On liver biopsy, sarcoidosis commonly shows nonnecrotizing epithelioid granulomas in the portal tract. They are generally larger than granulomas in other liver conditions and can coalesce over time. The granulomas contain multinucleated giant cells and show lymphocytic cuffing and fibrin depositions at the periphery.^1^^,^^9^ While histologic data is the best confirmatory method for diagnosing hepatic sarcoidosis, few studies have systematically reviewed liver histology. As in all forms of sarcoidosis, biopsies must be interpreted within a clinical context compatible with sarcoidosis. Most prior studies have included patients with isolated granulomatous hepatitis, including primary biliary cholangitis (PBC), idiopathic granulomatous hepatitis, drug-induced liver injury, and other systemic disorders that have different natural histories.^3^^,^^10^^,^^11^ Few studies have tried correlating liver histology with complications developing in hepatic sarcoidosis. The more severe manifestations of hepatic sarcoidosis include the development of portal HTN and cirrhosis, occurring in approximately 3%–18% of patients.^3^^,^^7^^,^^12^^,^^13^ The development of these complications is of great concern given the scant and sub-optimal treatment options available to treat the underlying disease process.^14^ Histological confirmation of hepatic sarcoidosis to support ATS guidelines can ultimately aid in its prognosis and help establish the need for medical treatment. Our study aimed to examine liver histology to assess disease severity better and help identify patients potentially at risk for developing portal HTN and cirrhosis.

## Methods

### Study Population and Data Collection

A retrospective cohort study was performed using the case finder program in Mount Sinai Hospital's Pathology department to screen for cases of hepatic sarcoidosis from 1994 to 2018 using the search criteria "sarcoidosis" and "liver." A list was compiled of all possible cases (n = 923) having a liver biopsy with a potential diagnosis of hepatic sarcoidosis. The case finder list was cross-referenced to a list containing all patients seen between 2014 and 2018 enrolled at Mount Sinai's Sarcoidosis Clinic or Liver Disease Department (n = 567). A total of 1490 patients was compiled from the 2 lists, but after accounting for repeat patients, a master list of patients (n = 1421) with the potential diagnosis of hepatic sarcoidosis was compiled.

The master list was refined and analyzed using Mount Sinai's electronic medical record system to categorize patients with sarcoidosis based on their specific organ involvement. Based on ATS criteria using instruments sponsored by the World Association of Sarcoidosis and Other Granulomatous Disorders some patients were excluded (n = 396) from the study or identified as not having sarcoidosis (n = 171).^15^ The remaining patients were categorized as having "possible sarcoidosis" (n = 25) or "confirmed sarcoidosis" (n = 829) in having the involvement of at least ≥2 organs. Patients were excluded from the analysis in the absence of extra-hepatic sarcoidosis. All patients in the “confirmed sarcoidosis” group met ATS diagnostic criteria for sarcoidosis.

**Figure 1:**
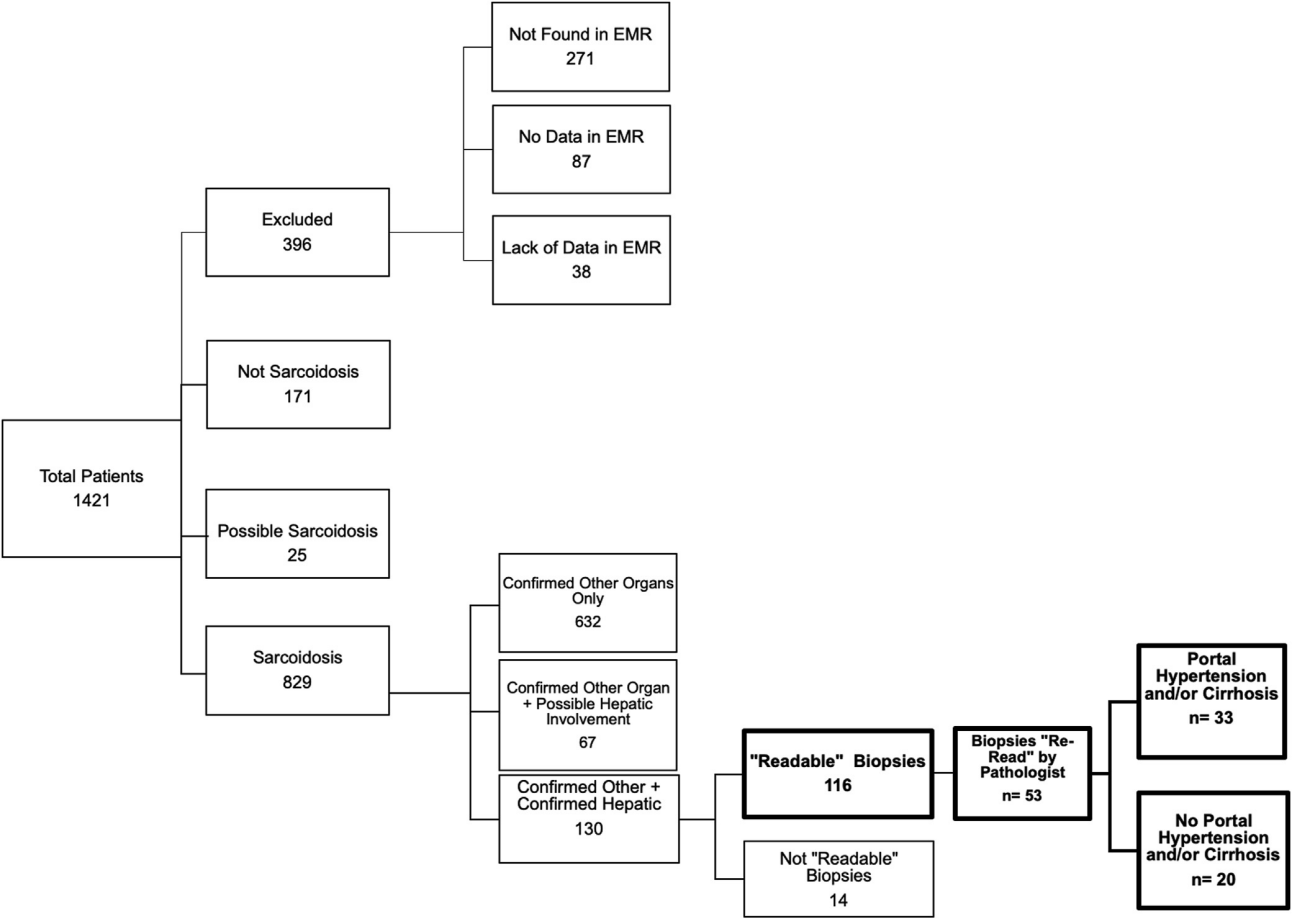
Flow chart depicting the study population selection process. The dark-bordered boxes show the final total study population and the specific groups the subjects were divided into. All confirmed cases of sarcoidosis met the American Thoracic Society (ATS) diagnosis criteria for sarcoidosis. Definite hepatic sarcoidosis cases had liver biopsy and clinical or radiologic findings consistent with diagnosis. Probable cases, those with liver biopsy and clinical and radiologic findings consistent with extra-hepatic sarcoidosis or cases including isolated granulomatous hepatitis, were excluded.

The "confirmed sarcoidosis" group was subdivided into the following groups: "Confirmed Other Organs" (n = 632), "Confirmed Other Organ + Possible Hepatic" (n = 67), and "Confirmed Other + Hepatic" (n = 130). Patients with possible hepatic sarcoidosis, including those with isolated granulomatous hepatitis, were excluded from the analysis. A detailed breakdown of the study population and criteria is shown in Figure 1.

### Data Collection

Patient demographics, abdominal imaging, laboratory testing, and liver biopsy results were reviewed. Data were collected for different time intervals, including at the time of diagnosis, treatment changes, and the most recent results. Abdominal imaging and esophagogastroduodenoscopy results were reviewed to assess for signs of cirrhosis and portal HTN, particularly for varices. There was no evidence of biliary obstruction when evaluating for portal HTN. Splenomegaly without the presence of varices on imaging or at esophagogastroduodenoscopy did not fulfill our criteria for portal HTN as sarcoidosis can directly involve the spleen.

### Histological Analysis

Archival liver biopsy slides were retrieved to reread the specimens, including hematoxylin and eosin, Masson trichrome, and CK7 immunostains. A score sheet tailored specifically for hepatic sarcoidosis was developed by an experienced liver pathologist (MIF). (See Supplementary 1).^16^, ^17^, ^18^ The histological analysis was conducted by the same liver pathologist who was blinded to the clinical data and independently reviewed the biopsies to ensure the uniform readability of all specimens. When interpreted within the proper clinical setting, the specimens that exhibited nonnecrotizing granulomas and other histopathologic features consistent with hepatic sarcoidosis were given a final determination of a confirmed diagnosis. The final study population was n = 53 unique patients. Furthermore, this histological analysis was further reviewed by dividing the cases into "portal hypertension and/or cirrhosis" ("pHTN+", n = 33 patients) vs "no portal hypertension and/or cirrhosis" ("pHTN-", n = 20 patients) groups. Any specimens that had confirmed histological features of hepatic sarcoidosis and other concomitant liver pathology, such as autoimmune hepatitis or tuberculosis based on case notes or histology, were excluded from the final analysis.

### Statistical Analysis

Data analysis was conducted by, first, utilizing Excel to organize the cases into their respective study groups: “total study population”, “pHTN+”, and “pHTN-”. Demographic and laboratory analyses were conducted amongst the final study groups utilizing t-test analysis. Statistical analyses comparing the histological findings between the pHTN+ and pHTN- groups were conducted for histologic feature evaluation. The features evaluated included granuloma characteristics (morphology, location, number), biliary characteristics (bile duct loss, bile duct damage, cholestasis, ductular reaction), fibrosis characteristics (staging), and inflammation (grade and type of inflammatory infiltrate). For these analyses, 2x2 and 2x4 contingency tables were developed to run Fisher’s exact test via the online data analysis tool VassarStat.^19^ All data were presented as total numbers, mean ± standard deviation, or percentages. Significance between all comparison groups was accepted at 2-tailed *P* values < 0.05.

## Results

### Demographics

Baseline patient characteristics were compared between the pHTN+ and pHTN- groups as shown in Table 1. In the pHTN + group, pulmonary (73%), lymph nodes (36%), spleen (21%), and skin (18%) were the most common other organs involved, whereas in the pHTN- group, it was pulmonary (75%), lymph nodes (35%), skin (15%), and ocular (15%)

**Table 1:** Baseline Characteristics Present Among the Portal Hypertension, Nonportal Hypertension, and Total Study Populations

Characteristics	Total (n = 53)	Portal hypertension and/or cirrhosis (n = 33)	No portal hypertension and/or cirrhosis (n = 20)	*P* value
Gender, % (n)				
Males	42	45	35	
Females	58	55	65	.45
Race, % (n)				
Hispanic, Latino, or Spanish	13	9	20	.40
American Indian	0	0	0	1.0
Asian	0	0	0	1.0
South Asian	4	6	0	.52
Black or African-American	42	45	35	.57
White	26	27	25	1.0
Native Hawaiian, Pacific Islander	0	0	0	1.0
Other	13	9	20	.40
Unknown	2	3	0	1.0
Age at diagnosis, mean ± SD				
In males	45 ± 11.8	51 ± 9.50	33 ± 6.53	.0004
In females	50 ± 8.63	49 ± 8.56	53 ± 8.50	.21
In total population	48 ± 10.3	49 ± 8.89	46 ± 12.2	
Other organ involvement, %				
Skeletal	2	3	0	
Eyes	8	3	15	
Lymph nodes	36	36	35	
Skin	17	18	15	
Heart	6	6	5	
Pulmonary	74	73	75	
Spleen	15	21	5	
Gastrointestinal	6	3	10	

The total study population (n = 53) was divided into 2 groups: those with portal hypertension and/or cirrhosis (pHTN+, n = 33) and those nonportal hypertension and/or cirrhosis (pHTN-, n = 20). Statistical significance analyses were performed for gender, race, and age at diagnosis. *P* value comparisons are between pHTN+ and pHTN- groups. (Chi Square, Fisher’s exact and 2-sample t-test, *P* < .05).

### Histological Comparison of Hepatic Sarcoidosis with and without Portal Hypertension and Cirrhosis

The biopsy specimens were divided into 2 groups (pHTN + or pHTN-) to compare any unique histologic features present amongst patients with hepatic sarcoidosis having more and less advanced histological disease (see Table 2). For biopsy samples evaluated, the pHTN + group had an average core length of 2.64 cm (range: 1.00–4.50 cm) and an average of 13 portal tracts (range: 5–20). The pHTN- group had an average core length of 2.55 cm (range: 0.90–3.80) and an average of 15 portal tracts (range: 4 to 48). Among both groups, all cases had nonnecrotizing epithelioid granulomas. No biopsies showed evidence of caseating necrosis, suppurative granulomas, fibrin rings, or lipo-granulomas. There were no statistical differences between the 2 groups (*P* = 1.0).

**Table 2:** Histologic Characteristics Present Among the Portal Hypertension and Nonportal Hypertension Study Populations

Histologic features	Total (n = 53)		Portal hypertension and/or cirrhosis (n = 33)		Nonportal hypertension and/or cirrhosis (n = 20)		*P* value
	Number	%	Number	%	Number	%	
Granuloma characteristics							
Morphology							
Noncaseating epithelioid granulomas	49	92.5	30	90.9	19	95.0	1.00
Caseating granulomas	0	0.0	0	0.0	0	0.0	1.00
Suppurative granulomas	0	0.0	0	0.0	0	0.0	1.00
Fibrin ring granulomas	0	0.0	0	0.0	0	0.0	1.00
Lipogranuloma	0	0.0	0	0.0	0	0.0	1.00
Location							
Portal	40	75.5	25	75.8	15	75.0	1.00
Periportal	25	47.2	13	39.4	12	60.0	.168
Lobular	33	62.3	18	54.5	15	75.0	.158
Number							
Few (1)	16	30.2	12	36.4	4	20.0	.237
Many (2)	10	18.9	5	15.2	5	25.0	.475
Confluent (3)	23	43.4	13	39.4	10	50.0	.570
Sclerosis	39	73.6	24	72.7	14	70.0	1.00
Sinusoidal dilation	24	45.3	16	48.5	8	40.0	.582
Variable age	28	52.8	15	45.5	13	65.0	.256
Biliary characteristics							
Bile duct loss							
Yes	19	35.8	16	48.5	3	15.0	**.0185∗**
Average % of BD loss	62.50%		61.69		59.5		
No	34	64.2	17	51.5	17	85.0	
Average % of BD loss	29.50%		35.59		21.11		
Cholestasis							
Yes	15	28.3	12	36.4	3	15.0	.123
No	38	71.7	21	63.6	17	85.0	
Bile duct damage							
Yes	39	73.6	28	84.8	11	55.0	**.0252∗**
No	14	26.4	5	15.2	9	45.0	
Ductular reaction							
None	16	30.2	6	18.2	10	50.0	**.0288∗**
Mild	34	64.2	24	72.7	10	50.0	.140
Moderate	2	3.8	2	6.1	0	0.0	.521
Severe	1	1.9	1	3.0	0	0.0	1.00
Fibrosis characteristics							
Staging							
0	7	13.2	2	6.1	5	25.0	.0896
1	16	30.2	6	18.2	10	50.0	**.0288∗**
2	13	24.5	9	27.3	4	20.0	.744
3	11	20.8	10	30.3	1	5.0	**.0371∗**
4	7	13.2	7	21.2	0	0.0	**.0365∗**
Biliary fibrosis							
Yes	13	24.5	11	33.3	2	10.0	.0978
No	40	75.5	22	66.7	18	90.0	
Inflammatory characteristics							
Type							
Mixed	2	3.8	2	6.1	0	0.0	.521
Plasma	11	20.8	6	18.2	5	25.0	.728
Lymphocytic	49	92.5	31	93.9	18	90.0	.00
Histiocytic	1	1.9	1	3.0	0	0.0	1.00
Eosinophilic	0	0.0	0	0.0	0	0.0	1.00
Portal inflammation							
0	6	11.3	1	3.0	5	25.0	**.0239∗**
1	42	79.2	28	84.8	14	70.0	.296
2	5	9.4	4	12.1	1	5.0	.638
3	0	0.0	0	0.0	0	0.0	1.00
4	0	0.0	0	0.0	0	0.0	1.00
Periportal inflammation							
0	42	79.2	23	69.7	19	95.0	**.0371∗**
1	9	17.0	8	24.2	1	5.0	.129
2	2	3.8	2	6.1	0	0.0	.521
3	0	0.0	0	0.0	0	0.0	1.00
Lobular inflammation							
0	19	35.8	13	39.4	6	30.0	.564
1	29	54.7	17	51.5	12	60.0	.582
2	5	9.4	3	9.1	2	10.0	1.00
3	0	0.0	0	0.0	0	0.0	1.00
Steatosis							
0	40	75.5	25	75.8	15	75.0	.599
1	10	18.9	6	18.2	4	20.0	.571
2	3	5.7	2	6.1	1	5.0	.684
3	0	0.0	0	0.0	0	0.0	1.00
Nodular regenerative hyperplasia (NRH)							
Yes	18	34.0	14	42.4	4	20.0	.136
No	35	66.0	19	57.6	16	80.0	
Venous outflow							
Yes	8	15.1	6	18.2	2	10.0	.471
No	45	84.9	27	81.8	18	90.0	
Autoimmune hepatitis histology							
Yes	0	0.0	0	0.0	0	0.0	1.00
No	53	100.0	33	100.0	20	100.0	
Chronic hepatitis							
Yes	13	24.5	10	30.3	3	15.0	.325
No	40	75.5	23	69.7	17	85.0	

*P* value comparisons of hepatic sarcoidosis patients with and without portal hypertension and/or cirrhosis (Fisher’s exact test, *P* < .05). Significant values are bolded and denoted with a ∗.

The portal, periportal, and lobular areas were noted for the presence of granulomas. The specimens in the pHTN + group showed granulomas in all 3 compartments. A single biopsy may have had granulomas present in multiple locations; however, the portal location was the most common (75.8%), and the lobular region was the second most common (54.5%). The pHTN- group also had granulomas within all 3 compartments. However, the portal and lobular regions were equally common regions for the presence of granulomas within this group (75%). There was no statistical difference when comparing the pHTN+ and pHTN- groups (*P* = 1.0, 0.17, 0.16, respectively). The total granuloma number was graded on a scale from 1 to 3, with 1 indicating few, 2 indicating many, and 3 indicating confluent. Among the pHTN + specimens, 36.4% had few, 15.2% had many, and 39.4% had confluent granulomas. Among the pHTN- specimens, 20% had few, 25% had many, and 50% had confluent granulomas. The number of granulomas was variable amongst both analysis groups and showed no statistical differences (*P* = .24, .48, 0.57, respectively). There was sinusoidal dilation in 16 pHTN + specimens and 8 pHTN- specimens (*P* = .58). Fifteen pHTN + specimens and 13 pHTN- specimens showed granulomas of varying ages (*P* = .26).

Fibrosis was staged based on a scale of 0 to 4.^20^ Between the 2 groups, the pHTN- group had a higher percentage of patients with fibrosis between stages 0 and 1 (25% and 50%, respectively) compared to the pHTN + group (6.1% and 18.2%, respectively). The biopsies from patients with pHTN + had predominantly stage 3 (30.3%) and stage 4 (21.2%) fibrosis compared to the pHTN- group, which only showed one biopsy with stage 3 fibrosis and zero with stage 4 (*P* = .037 for stage 3 and *P* = .037 for stage 4). While the pHTN + group had a greater number of biopsies showing features of biliary fibrosis, this difference was not statistically different compared to the pHTN- group (*P* = .098).

Analysis showed that the liver biopsies predominantly showed lymphocytic inflammation (93.9% in the pHTN + group and 90% in pHTN-). The next most common type of inflammation was plasmacytoid (18.2% in the pHTN+ and 25% in the pHTN- groups). Rare biopsies from the pHTN + group also showed mixed (6.1%) and histiocytic (3%) types of inflammation, whereas the pHTN- group did not. Inflammation in the portal, periportal, and lobular regions was graded. Inflammation in the portal region was staged on a scale from zero to four.^20^ Amongst both study populations, portal inflammation was found only up to a severity of grade 2, with the highest percentage graded as grade 1 (84.8% for pHTN+ and 70% for pHTN-). When comparing pHTN+ and pHTN- groups, inflammation within the portal region showed a statistical difference for grade 0 (*P* = .024). Periportal and lobular inflammation were graded on a scale from zero to 3. Comparison of periportal inflammation revealed 23 patient specimens with grade 0 in the pHTN + group and 19 in the pHTN- group (*P* = .037). Eight biopsies were grade 1 in the pHTN + group vs one in the pHTN- group (*P* = .13). Two patient biopsies were grade 2 in the pHTN + group and none in the pHTN- group (*P* = .52). No group showed grade 3 periportal inflammation. Lobular inflammation showed similar results with biopsies from both groups largely grade 0 (n = 13 in pHTN + vs n = 6 in pHTN-, *P* = .35) and grade 1 (n = 17 in pHTN + vs n = 12 in pHTN-, *P* = .564). A limited number of patient biopsies were grade 2 lobular inflammation (n = 3 for pHTN+ and n = 2 for pHTN-, *P* = 1.0). No specimens showed grade 4 lobular inflammation.

Compared to the pHTN- group, the pHTN + group had double the percentage of nodular regenerative hyperplasia (NRH) (Table 2, 42.4% in pHTN+ and 20% in pHTN-, *P* = .14). Venous outflow obstruction was observed in 18.2% of the patient biopsies in the pHTN+ and 10% of the pHTN- group (*P* = .47). No biopsy from either study group showed evidence of autoimmune hepatitis. However, about 30% of the pHTN + patient biopsies showed chronic hepatitis as compared to only 15% in the pHTN- group (*P* = .33).

**Figure 2:**
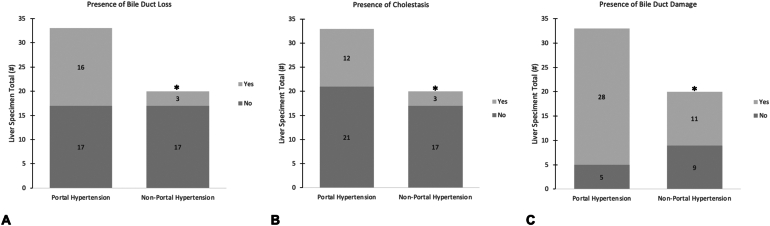
Total number of liver biopsies showing bile duct loss, cholestasis, and bile duct damage among patients with hepatic sarcoidosis with and without portal hypertension and/or cirrhosis. Histologic analysis was conducted on 53 liver biopsies, further divided into portal hypertension (n = 33) and nonportal hypertension (n = 20). (A) shows the total number of liver specimens with the presence or absence of bile duct loss (defined by greater than 50% loss). (B) shows the total number of liver specimens showing evidence or absence of cholestasis. (C) shows the total number of liver specimens with the presence or absence of bile duct damage. Statistical analysis compared the presence of biliary characteristics discussed above between the portal and nonportal hypertension groups. The light gray boxes represent those showing evidence of each histologic feature. The dark gray box represents those that did not. Statistically different values between the 2 study groups are denoted with an ∗ (Fisher’s exact test, *P* < .05).

Histologic biliary features documented in previous studies, including bile duct loss, cholestasis, and bile duct damage, were also compared among the pHTN+ and pHTN- groups.^17^^,^^18^ Figure 2 depicts the findings observed between the 2 groups. Sixteen patients in the pHTN + group showed at least 50% bile duct loss compared to 3 patients in the pHTN- group (Figure 2A, *P* = .019). In the pHTN + group, the 16 biopsies with bile duct loss showed an average bile duct loss of 62% (range 50%–85%). In the 3 biopsies in the pHTN- group bile duct loss was on average 60% (range 57%–82%). Figure 2 also compares the presence of cholestasis between the study populations. The pHTN + group had 12 biopsies with cholestasis, and the pHTN- group had 3. Statistical difference was observed when comparing bile duct damage between the 2 study populations. The pHTN + group revealed 28 liver biopsies with bile duct damage, whereas the pHTN- group had 11 (Figure 2C, *P* value = .025). Eight patients in the pHTN + group had some degree of outflow obstruction which could be contributing to the portal HTN, whereas 2 in the pHTN- group had venous outflow obstruction.

The final component of the pathology review analyzed the presence and severity of ductular reaction. Analysis was first conducted between pHTN+ and pHTN- groups, comparing the different degrees of ductular reaction (none, mild, moderate, severe). This analysis revealed a *P* value of 0.045. This likely reflects the concurrent biliary damage occurring predominantly in the pHTN + group. Ten patients from the pHTN- group did not show signs of ductular reaction compared to 6 in the pHTN + group (*P* = .029). Interestingly, a total of 24.6% of patients also had some degree of steatosis, which itself in some cases could confound the typical abnormal liver biochemistries noted in patients having hepatic sarcoidosis.

### Biliary Cirrhosis

A further analysis was conducted of the pHTN + group differentiating cases based on the presence of fibrosis: minimal fibrosis (stage 0 and 1, n = 8) vs advanced fibrosis (stage 3 and 4, n = 17) and minimal fibrosis (stage 0 and 1) vs cirrhosis (stage 4, n = 7). There were 7 cases that revealed cirrhosis, with all showing histologic evidence of biliary cirrhosis, apart from one. Biliary-type fibrosis was characterized by portal tracts devoid of inflammation but prominent proliferating ductules. In biliary cirrhosis, disease progression causes chronic changes such as broad fibrous septa development from portal tracts, and periseptal hepatocytes are often swollen with granular copper deposition consistent with cholestasis. The native interlobular bile ducts are either lost or replaced by fibrosis, with irregular nodules surrounded by dense fibrous septa forming at later stages. Table 3 compares various histologic features amongst the pHTN + group having minimal fibrosis, advanced fibrosis, and biliary cirrhosis. Expectedly, the presence of biliary fibrosis was greater within the advanced fibrosis (53%, *P* = .0088) and cirrhosis (71%, *P* = .041) groups compared to the minimal fibrosis group (13%). Interestingly, the number of granulomas did not produce a statistical difference between the groups. The minimal fibrosis group had 25% with few granulomas, 38% with many, and 25% with confluent granulomas. The advanced fibrosis group had 47% with few granulomas (*P* = .40), 6% with many (*P* = .081), and 35% confluent (*P* = .68). The cirrhosis group had 29% with few granulomas (*P* = 1.0), 15% with many (*P* = .57), and 43% confluent (*P* = .61, Table 3).

**Table 3:** Histologic Comparisons Amongst Portal Hypertension Group With Varying Degrees of Fibrosis

Histologic features	Minimal fibrosis (n = 8)		Advanced fibrosis (n = 17)		Cirrhosis (n = 7)		*P* value
	Number	%	Number	%	Number	%	
Granuloma characteristics							
Morphology							
Noncaseating epithelioid granulomas	7	88	15	88	6	86	1.0, 1.0
Caseating granulomas	0	0	0	0	0	0	
Suppurative granulomas	0	0	0	0	0	0	
Fibrin ring granulomas	0	0	0	0	0	0	
Lipogranuloma	0	0	0	0	0	0	
Location							
Portal	5	63	12	71	6	86	1.0, .569
Periportal	2	25	7	41	4	57	.661, .231
Lobular	4	50	7	41	3	43	1.0, 1.0
Number							
Few (1)	2	25	8	47	2	29	.402, 1.0
Many (2)	3	38	1	6	1	14	.0808, .569
Confluent (3)	2	25	6	35	3	43	.680, .608
Biliary characteristics							
Bile duct loss							
Yes	2	25	11	65	5	71	.0968, .132
Average % of BD loss		65		61	60		
No	6	75	6	35	2	29	
Average % of BD loss		31		24	29		
Cholestasis							
Yes	2	25	8	47	4	57	.402, .315
No	6	75	9	53	3	43	
Bile duct damage							
Yes	7	88	16	94	6	86	1.0, 1.0
No	1	13	1	6	1	14	
Ductular reaction							
None	2	25	1	6	1	14	.231, 1.0
Mild	6	75	13	76	5	71	1.0, 1.0
Moderate	0	0	2	12	1	14	.547, .467
Severe	0	0	1	6	0	0	1.0, 1.0
Biliary fibrosis							
Yes	1	13	9	53	5	71	.0875, **.0406∗**
No	7	88	8	47	2	29	
Inflammatory characteristics							
Type							
Mixed	0	0	1	6	1	14	1.0, .467
Plasma	0	0	5	29	2	29	.140, .2
Lymphocytic	8	100	16	94	6	86	1.0, .467
Histiocytic	0	0	1	6	0	0	1.0, 1.0
Eosinophilic	0	0	0	0	0	0	1.0, 1.0
Portal inflammation							
0	0	0	1	6	0	0	1.0, 1.0
1	7	88	13	76	5	71	.642, .569
2	1	13	3	18	2	29	1.0, .569
3	0	0	0	0	0	0	1.0, 1.0
4	0	0	0	0	0	0	1.0, 1.0
Periportal inflammation							
0	7	88	9	53	5	71	.182, .569
1	0	0	7	41	1	14	.0573, .467
2	1	13	1	6	1	14	1.0, 1.0
3	0	0	0	0	0	0	1.0, 1.0
Lobular inflammation							
0	6	75	7	41	2	29	.201, .132
1	1	13	1	6	4	57	1.0, 0.119
2	1	13	2	12	1	14	1.0, 1.0
3	0	0	0	0	0	0	1.0, 1.0

The portal hypertension group (n = 33) was divided into 3 groups based on stages of fibrosis: minimal (stage 0 and 1), advanced (stage 3 and 4), and cirrhosis (stage 4). A variety of histologic features including granulomas, fibrosis, and inflammation were reviewed. *P* value comparisons are between the minimal and advanced fibrosis groups and minimal and cirrhosis groups, respectively (Fisher’s exact test, *P* < .05). Significant values are denoted with a ∗.

BD, bile duct.

### Laboratory Data

Laboratory data at the time of liver biopsy along with the most recent (average 4–6 years after initial laboratory data) were compared for the pHTN+ (n-33) and pHTN- (n = 20) groups (see Supplementary 2). For the pHTN + group, the mean platelet value at diagnosis was 172 ± 75 and for the pHTN- group was 262 ± 90 (*P* = .004). There was no significant difference between the study groups for the other laboratory values at diagnosis.

Comparison of laboratory data that were obtained on most recent follow-up between the 2 groups showed a difference in multiple laboratory markers. The mean angiotensin-converting enzymelevel for the pHTN + group was 108 and for the pHTN- group was 29 (*P* = .009). The mean platelet levels were lower for the pHTN+ (137 ± 83) compared to the pHTN- group (214 ± 108, *P* = .018). Albumin was also lower in the pHTN + group (3.4 ± 0.7) compared to the pHTN- group (4.0 ± 0.8, *P* = .008). Average ALP levels were about 1.5x more elevated in the pHTN + group (245 ± 156) compared to the pHTN- group (155 ± 121, *P* = .049). The average values for total bilirubin, aspartate aminotransferase, alanine transaminase (ALT), and GGT showed no significant difference between the groups.

Laboratory data within each study group were also compared. For the pHTN + group, ALP was about 1.5x more at diagnosis (370 ± 238) compared to the most recent value (245 ± 156) (*P* = .030). Similarly, GGT was significantly elevated at diagnosis (725 ± 545) compared to the most recent values (294 ± 333, *P* = .022). Albumin was higher at time of diagnosis (3.7 ± 0.4) compared to the most recent labs (3.4, *P* = .051), but without statistical difference. For the pHTN- group, both ALT and ALP decreased from time of diagnosis to most recent. ALT at diagnosis (92 ± 97) was twice the most recent value (39 ± 26, *P* = .046). Similarly, ALP at diagnosis was 349 ± 311; about 2 times the most recent value: 155 ± 121 (*P* = .031). No other laboratory values showed significant difference within the pHTN- group. The mean platelet count at time of liver biopsy in the pHTN + group was 172 x 10^9^/L, thus making it a suboptimal surrogate marker for diagnosing portal HTN, especially since decreased platelet counts can be related to splenic involvement by sarcoidosis in the absence of portal HTN.

### Treatment Data

**Figure 3:**
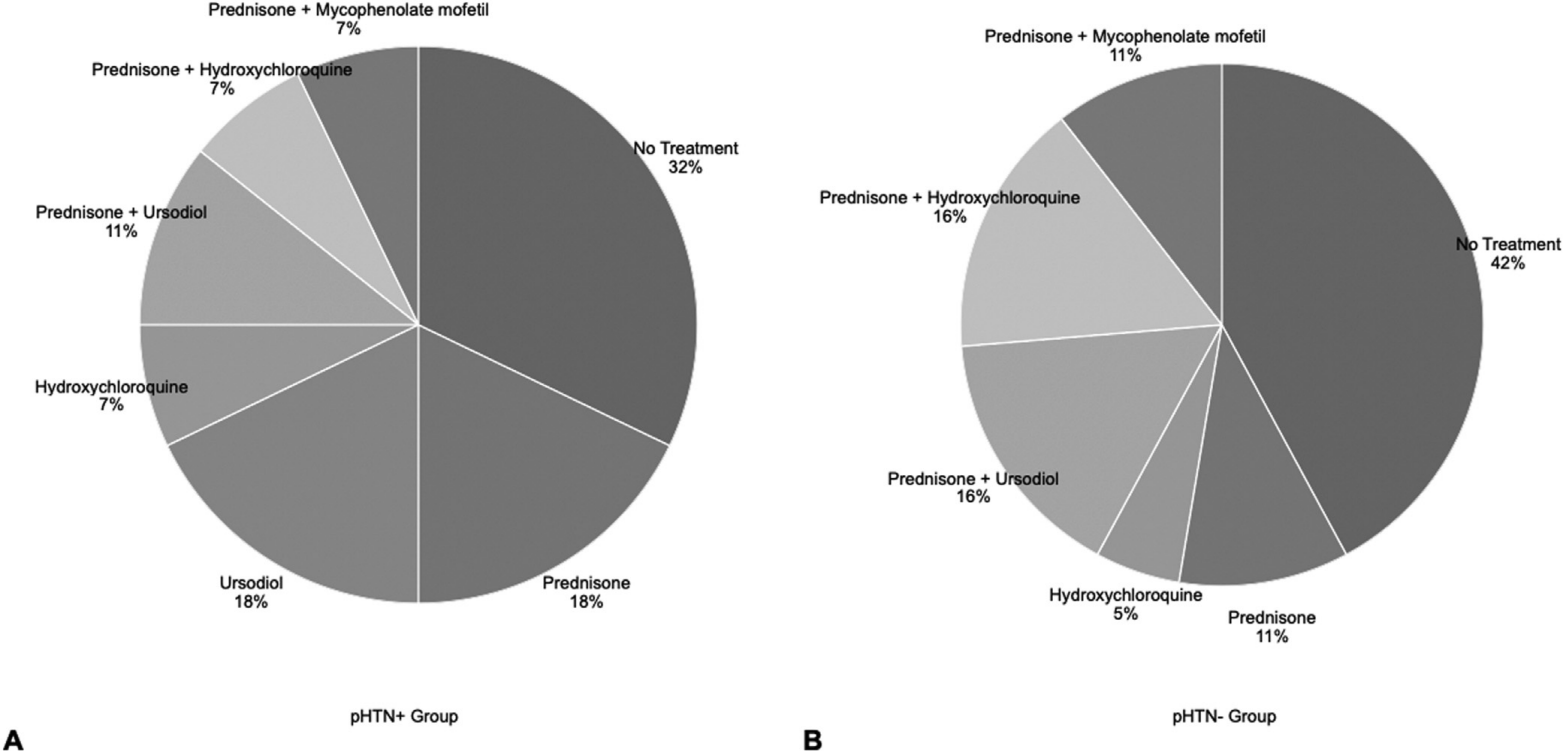
Treatments administered to patients with hepatic sarcoidosis with and without portal HTN and/or cirrhosis. (A) shows treatments for pHTN + group (n = 33). (B) shows treatment for pHTN- group (n = 20). Treatments administered are represented in percentages (%). Not displayed in the figure are cases (pHTN + groups, n = 5 and pHTN- n = 2) for which treatment data were unavailable in the chart. Specific timelines of treatment durations are variable and dependent upon each case and were not evaluated as part of this study due to data availability limitations. On average, however, there was a 4 to 6-year period between the initial diagnosis and the most recent labs.

Sarcoidosis treatment data for the pHTN + group and pHTN- groups are shown in Figure 3, comparing the treatments administered at initial diagnosis and liver biopsy up to the point when the most recent laboratory data were collected.

### Patients Having Multiple Biopsies (n = 7)

Amongst the total study population (n = 53), there were 7 patients who underwent multiple liver biopsies. A separate review and comparison of the patients with multiple biopsies was conducted. Comparison was particularly drawn between the biopsy at time of diagnosis and the most recent biopsy. On average biopsies were taken about 1.5 years apart (Table 4). Some trends appreciated through this analysis included a change in granuloma distribution from the first biopsy to the most recent. For example, at time of diagnosis, 86% of granulomas were in the portal location, 29% in periportal, and 14% in lobular. However, on repeat biopsy, 43% were in the periportal region and 29% in the lobular region. Similarly, at time of diagnosis, 86% had mild degree of ductular reaction and 14% had moderate. However, on repeat biopsy, 29% had evidence of severe ductular reaction. A similar increase in presence of bile duct loss and cholestasis was appreciated between the diagnostic biopsy and most recent. However, these changes were not statistically significant.

**Table 4:** Summary of Patients With Multiple Biopsies in Both the Portal Hypertension and Nonportal Hypertension Groups

Patient (n = 7)	Number of biopsies	Average time between each biopsy	Liver chemistry tests of initial and most recent biopsy								Treatment initiated	Transplanted	Deceased
			AST (U/L)	ALT (U/L)	ALP (U/L)	Total bilirubin (mg/dL)	Albumin (g/dL)	Platelets (10^9^/L)	INR	GGT (U/L)			
Patient 1	2	1 y	68	88	500	3.4	2.9	204	1.1	1055	UDCA 200 mg daily	Yes	No
			Not available										
Patient 2	5	9 mo	180	205	440	0.7	3.7	190	1.0	Not available	Not available	Yes	Yes
			76	33	175	12.8	2.6	32	10.5	52	Not available		
Patient 3	2	2 y	131	73	394	1.1	2.7	127	Not available	324	Not available	Yes	Yes
			111	66	493	1	2.6	92	Not available	482	Not available		
Patient 4	3	6 mo	Not available								Not available	Yes	No
			Not available								Not available		
Patient 5	2	3 y	42	66	462	1.1	4	Not available	Not available	562	Not available	No	No
			49	34	196	4.3	2.8	77	1.4	272	Prednisone 10 mg daily		
Patient 6	2	3 y	21	19	157	0.3	4.3	244	0.9	Not available	Prednisone 5 mg daily hydroxychloroquine 400 mg daily	Yes	Yes
			23	6	263	0.5	2.7	263	1.1	Not available	UDCA 900 mg daily mycophenolate mofetil 500 mg twice daily		
Patient 7	2	1 y	24	54	406	0.9	3.5	8	1.1	1261	Prednisone 60 mg daily	No	Yes (other)
			20	37	224	541	4.5	19	1.3	541	Not available		

Cases include both patients in the pHTN+ (n = 5) and pHTN- (n = 2) groups. Data show laboratory data (initial (first line) and most recent (second line)) for platelets, INR, total bilirubin, albumin, aspartate aminotransferase (AST), alanine transaminase (ALT), alkaline phosphatase (ALP), gamma-glutamyl transpeptidase (GGT). *P* value comparisons were not conducted for this descriptive representation.

UDCA, Ursodeoxycholic acid.

## Discussion

Hepatic sarcoidosis most commonly presents with abnormal liver chemistry test abnormalities in the background of well-established extra-hepatic and/or systemic disease. However, some patients go on to develop portal HTN and cirrhosis, consistent with advanced disease. Liver histology may be invaluable in aiding clinicians to understand the pathophysiology of these more severe forms of hepatic sarcoidosis. There are limited published data examining liver histology in sarcoidosis, with previous studies likely enriched with patients having nonsarcoid granulomatous hepatitis.^21^, ^22^, ^23^ Few studies of hepatic sarcoidosis have been conducted that definitively and reliably exclude other granulomatous diseases such as PBC.^24^ We investigated the histological characteristics for one of the largest hepatic sarcoidosis cohorts to date, and further analyzed the patients having more severe forms with portal HTN or cirrhosis.

In the current study the main observations are as follows: (1) patients with hepatic sarcoidosis having portal HTN have greater degrees of biliary fibrosis, compared to those without portal HTN; (2) hepatic sarcoidosis is a cause of biliary cirrhosis which may develop seemingly through direct insult to bile ducts; (3) a distinguishing characteristic in patients having portal HTN/cirrhosis is the presence of bile duct loss; and (4) the level of biliary insult and its association with advanced stages of fibrosis potentially suggests a different pathophysiology for the disease rather than the presumed ongoing parenchymal damage over time related to granulomatous inflammation. Within this context, treatment with systemic anti-inflammatory therapy may be contraindicated in certain patients. Patients in the pHTN + group had NRH, advanced fibrosis, or venous outflow obstruction present on liver biopsy, all of which could account for the clinical portal HTN. It is possible that the small number of patients having NRH or outflow obstruction in the pHTN- group had yet to develop elevated portal pressures but might do so in the future.

### Fibrosis Patterns in Hepatic Sarcoidosis

Biopsies obtained from patients with hepatic sarcoidosis having portal HTN displayed higher stages of fibrosis (stage 3 (30%) and stage 4 (21%)) compared to cases of nonportal hypertention (stage 3 5% and stage 4 0%, respectively). Devaney et al reviewed the clinico-pathologic features of 100 patients, of which 21% exhibited a significant degree of fibrosis. The majority were limited to the portal and periportal regions and 6 cases showed cirrhosis. Patients with isolated granulomatous hepatitis were not excluded from the study, and it was noted that 3% of the study population had portal HTN.^7^

Our current study is unique because our study population (n = 53) included a large number of patients having portal HTN (62%, n = 33). Furthermore, our cohort included patients with portal HTN due to both noncirrhotic and cirrhotic causes. Several mechanisms causing portal HTN and cirrhosis in these patients have been postulated. Some of these mechanisms include the following: (1) increased portal flow and intra-hepatic pressure caused by arterial-venous shunts in areas of granulomas and fibrosis, (2) vascular restructuring (NRH) within the parenchyma secondary to increased scarring and fibrosis, (3) increasing portal pressures caused by presinusoidal obstruction and destruction (partial or complete) of portal venules, (4) bile duct damage or destruction causing resistance to blood flow, liver architectural distortion, and secondary biliary cirrhosis, and (5) development of outflow obstruction and increased postsinusoidal flow resistance secondary to scarring, granulomas, and inflammation.^13^

Although previous publications show that NRH can contribute to portal HTN, a biopsy is necessary to delineate the cause of portal HTN better. Our study demonstrates that NRH may be the cause of portal HTN and a cirrhotic appearing liver on imaging studies in some patients having hepatic sarcoidosis. Within the pHTN + group, 18 patients had NRH. While the difference in the presence of NRH is not significant, the presence is early and could determine later development. Thus, clinicians should consider liver biopsy more often in patients suspected to have hepatic sarcoidosis to help counsel patients regarding treatment and prognosis. Noninvasive fibrosis assessments have not been found to be an accurate assessment tool in patients with hepatic sarcoidosis.^16^^,^^25^, ^26^, ^27^, ^28^, ^29^ NRH occurs in conditions that are associated with perfusion abnormalities such as Fontan associated liver disease, as well as in inflammatory conditions not having hepatic inflammation such as in collagen vascular disease or in thrombotic disorders.^30^^,^^31^ The most likely etiology is a hepatic vascular endothelial insult that can similarly occur in hepatic sarcoidosis. Venous outflow obstruction may also be a result of vascular injury, which was demonstrated in 15% of the study population. The current study demonstrates that biliary cirrhosis may be the end result of hepatic sarcoidosis. Chronic liver diseases, such as PBC and primary sclerosing cholangitis that damage bile ducts may progress to biliary fibrosis. In these conditions, biliary fibrosis begins in the portal tracts and expands to the lobular area, eventually leading to progressive fibrosis and biliary cirrhosis.^32^^,^^33^ Biliary fibrosis and chronic cholestatic disease have been noted in hepatic sarcoidosis with an elevated ALP level as the most common biochemical abnormality.^34^ In the present study, the location and number of granulomas did not seem to impact severity of disease. For example, between the 2 study groups, the pHTN + group did not have a greater number of confluent granulomas compared to the pHTN- group. Thus, the biliary damage may be unrelated to parenchymal granulomatous inflammation.

### Bile Duct Loss as a Distinguishing Characteristic on Histology

Biliary cirrhosis resulting from sarcoidosis has been infrequently reported.^7^ In the present study, there were 7 cases of cirrhosis and of these, 6 showed bile duct damage and bile duct loss. Of the 6 that showed bile duct damage, 2 cases had 27% and 30% bile duct loss respectively, thus not meeting histologic criteria to classify as bile duct loss. The one case that did not show bile duct damage had 85% bile duct loss. There is precedent for other causes of bile duct loss to have a vascular etiology such as that occurs with chronic ductopenic rejection postliver transplantation or with biliary ischemia leading to secondary biliary cirrhosis. Whether a hepatic vascular endothelial injury contributes to the biliary findings noted in sarcoidosis warrants further study, especially with the frequent concurrent finding of NRH, which itself is related to a vascular remodeling in the liver.^31^^,^^35^^,^^36^

It is possible that the cases of biliary cirrhosis were end stage and “burnt out”, accounting for the lack of concurrent inflammation, and that granulomatous inflammation led to the biliary damage when present much earlier in the clinical course. However, the lack of significant inflammation also in the NRH group suggests they are unrelated. NRH is typically accompanied only by mild portal inflammation and can be found concurrently in PBC and primary sclerosing cholangitis.^36^, ^37^, ^38^, ^39^ Granulomatous vasculitis has been noted in sarcoidosis, which thus raises the possibility of a hepatic vasculitic process contributing to the biliary cirrhosis.^7^^,^^12^^,^^13^^,^^40^

Based on our current data the presence of bile duct damage and loss may be more concerning for the future development of portal HTN compared to merely the presence of granulomas and inflammation. In the current study, a similar pattern of granulomas and inflammation was noted across the range of biopsies, including similarly in the absence or presence of portal HTN and cirrhosis. Our data raise the possibility that if there are signs of bile duct damage and loss seen on biopsy, this may itself be a risk factor for the future development of biliary cirrhosis. Thus, in the presence of bile duct damage more aggressive treatment or closer follow-up with repeat liver biopsy may be warranted.

### Inflammation Patterns in Hepatic Sarcoidosis on Histology

The current study demonstrates that in both pHTN+ and pHTN- groups, inflammation similarly occurs in the lobular, portal, and periportal regions. Additionally, the degree of inflammation primarily exists at grade 0 or 1 for both groups. The pHTN + group had a slightly higher percentage of biopsies exhibiting grade 2 inflammation, particularly in the portal and periportal regions. However, this difference was not statistically different. Furthermore, in neither of the groups was grade 3 periportal inflammation noted. This suggests that granulomatous inflammation, let alone a greater degree of inflammation, may not be the major or sole contributor to the development of cirrhosis and portal HTN in this population.

Limited prospective data exist on the use of corticosteroids or other treatments for managing hepatic sarcoidosis. Some data have observed that most patients do not experience significant improvement in biochemical markers with treatment. However, Bakker et al. did appreciate an improvement in liver enzymes, including aspartate aminotransferase, ALT, ALP, and GGT levels after 3 months of treatment with ursodeoxycholic acid.^10^ Similarly, the present study noted improvement in liver chemistries, particularly ALP, from time of onset to about 4–6 years. Patients were treated with different therapies, with about 25% in both study groups receiving dual therapy including corticosteroids and an additional agent such as ursodeoxycholic acid, hydroxychloroquine, or mycophenolate mofetil. We could not find any correlation between the use of medical therapy and histologic improvement in the small number of patients having paired liver biopsies.

The present study is one of the largest histologic hepatic sarcoidosis studies to date, with one of the most stringent inclusion criteria that exclude patients having other granulomatous liver disorders such as PBC, forms of drug-induced liver injury, infections, and idiopathic granulomatous hepatitis.^3^^,^^7^^,^^22^ Utilizing strict inclusion and exclusion criteria ensured maximizing the chances of a study population with hepatic sarcoidosis only and no other confounding liver disease. None of the study patients had isolated hepatic sarcoidosis, which potentially might have a different natural history from the current cohort. Another strength of the current study is that although it is retrospective, we conducted a re-evaluation of all biopsy samples that were selected after going through a very meticulous and stringent inclusionary and exclusionary review. This blinded review was conducted by an experienced liver pathologist from a major academic center having a large, long standing sarcoidosis clinic.

Our retrospective study has limitations. The number of our study samples within the pHTN+ and pHTN- groups is relatively small, which creates difficulty in determining statistical significance. It is important to recognize that though the prevalence of hepatic sarcoidosis is not common, the true prevalence is probably under-estimated given that most studies for the disease so far have been retrospective, and most patients have sub-clinical disease with liver involvement often being found incidentally. For instance, abnormal liver chemistry tests have been reported to occur in as many as 20%–40% of patients having sarcoidosis, the majority of who have very mild disease.^11^ Our study was not meant to assess cases of isolated hepatic sarcoidosis and whether it is a true entity or what might be its natural history. There is clearly some selection bias present with the decision of the clinician to perform the liver biopsy as they were not done in a protocolized fashion. All patients in the pHTN + group had NRH, advanced fibrosis, or outflow obstruction that would account for portal HTN. A liver biopsy is, thus necessary to ascertain whether cirrhotic or noncirrhotic portal HTN is present to understand the potential risks for future liver decompensation and the risk for hepatocellular carcinoma development.

In summary, our findings demonstrate that patients with hepatic sarcoidosis had NRH, outflow obstruction, and advanced biliary fibrosis as the etiology for their portal HTN. There was no correlation with the degree or extent of granulomatous inflammation. Our findings suggest that the presence of bile duct loss or damage on liver biopsy might identify patients with a greater risk of progression of their liver disease. Although biliary abnormalities exist, it is unknown to what degree granulomatous inflammation contributes to biliary duct damage, further emphasizing why a biopsy is needed. We recommend the consideration of liver biopsy in patients having sarcoidosis if there is evidence of portal HTN to differentiate the presence of noncirrhotic portal HTN vs advanced biliary fibrosis and cirrhosis. Direct bile duct damage and loss rather than granulomatous inflammation (which is difficult to determine by current methods of testing, including FDG-PET scanning) may be the histologic triggers for more aggressive disease, and thus their presence on liver biopsy should be considered in the decision-making process whether to initiate medical therapy. More studies are needed to assess the role of liver biopsy in sarcoidosis, including that of paired biopsies, in order to explore the potential merits of medical treatment for which scant published data currently exist.

## Footnotes

Material associated with this article can be found in the online version at https://doi.org/10.1016/j.gastha.2024.10.001.
