# Supplementary material for: The Varying Histology of Hepatic Sarcoidosis and the Relation of Bile Duct Damage and Loss to the Presence of Portal Hypertension and Cirrhosis
Source: Gastro Hep Adv. 2024 Oct 10;4(2):100561. doi: 10.1016/j.gastha.2024.10.001 (PMC11757786; doi:10.1016/j.gastha.2024.10.001)
Supplement: Supplementary 1 [file mmc1.docx]

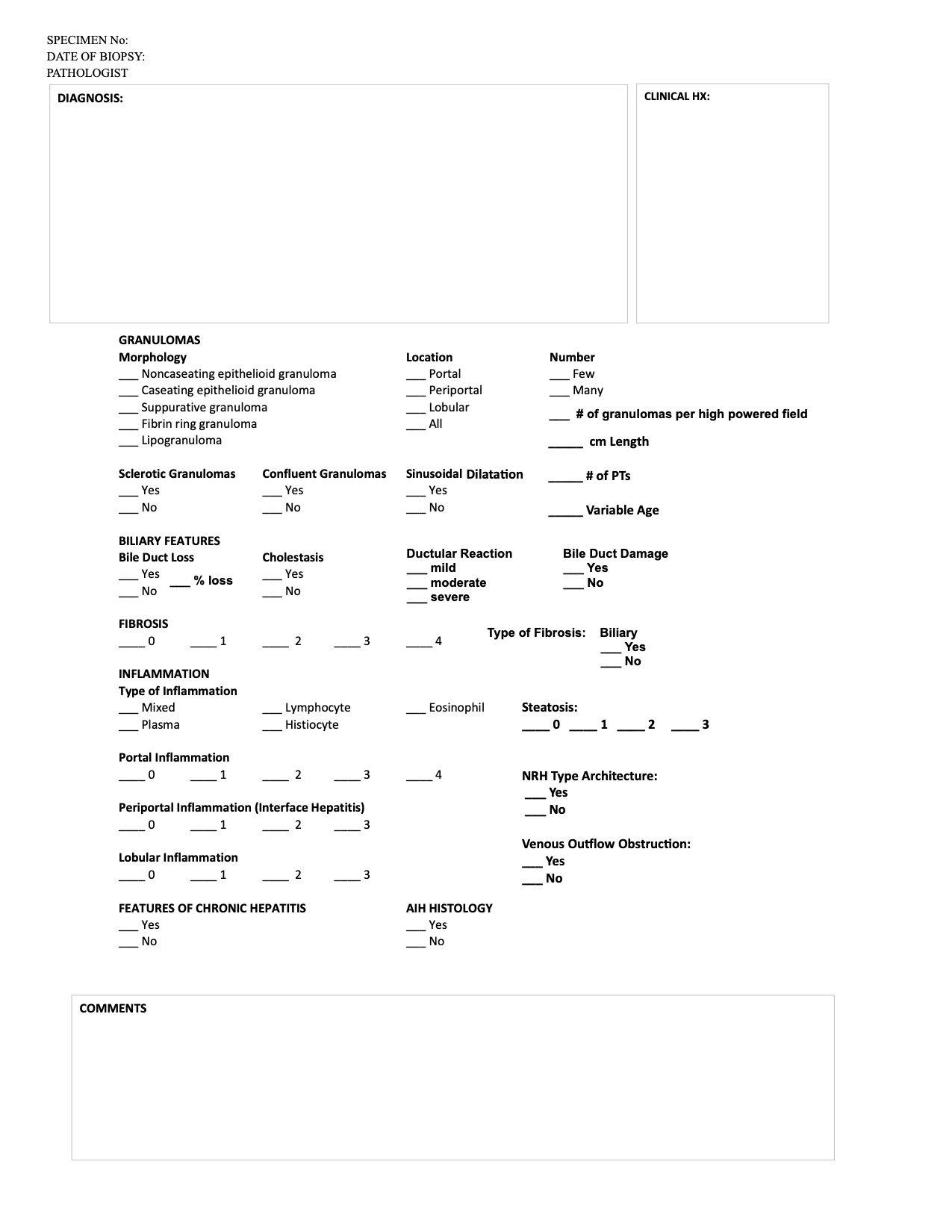


**Supplementary 1** *Scoring sheet for histologic evaluation for hepatic sarcoidosis*

Scoring sheet developed by a liver pathologist (MIF) for the review of histologic slides of hepatic sarcoidosis. Scoring sheet includes criteria commonly used to evaluate cases of granulomatous diseases as well as features that are commonly observed in hepatic sarcoidosis.
