# Supplementary material for: The Varying Histology of Hepatic Sarcoidosis and the Relation of Bile Duct Damage and Loss to the Presence of Portal Hypertension and Cirrhosis
Source: Gastro Hep Adv. 2024 Oct 10;4(2):100561. doi: 10.1016/j.gastha.2024.10.001 (PMC11757786; doi:10.1016/j.gastha.2024.10.001)
Supplement: Supplementary 2 [file mmc2.docx]

|  | **At Diagnosis** | | | | |  | **Portal HTN Group** | | | | |
| --- | --- | --- | --- | --- | --- | --- | --- | --- | --- | --- | --- |
| **Laboratory Data** | **Portal HTN (n= 33)** | | **No Portal HTN (n =20)** | |  | **Laboratory Data** | **Diagnosis** | | **Most Recent** | |  |
|  | **Value (Mean)** | **STD** | **Value (Mean)** | **STD** | **P-value** |  | **Value (Mean)** | **STD** | **Value (Mean)** | **STD** | **P-value** |
| ACE (nmol/mL/min) | 74 | 35 | 49 | 55 | 0.33 | ACE (nmol/mL/min) | 74 | 35 | 108 | 27 | 0.12 |
| Platelets (10^9^/L) | 172 | 75 | 262 | 90 | **0.004** | Platelets (10^9^/L) | 172 | 75 | 137 | 83 | 0.146 |
| Total Bilirubin (mg/dL) | 1.1 | 1 | 1 | 1.1 | 0.713 | Total Bilirubin (mg/dL) | 1.1 | 1 | 2.1 | 17 | 0.129 |
| Albumin (g/dL) | 3.7 | 0.4 | 3.8 | 0.7 | 0.731 | Albumin (g/dL) | 3.7 | 0.4 | 3.4 | 2.9 | 0.051 |
| AST (U/L) | 60 | 40 | 75 | 75 | 0.474 | AST (U/L) | 60 | 40 | 54 | 33 | 0.551 |
| ALT (U/L) | 62 | 45 | 92 | 97 | 0.251 | ALT (U/L) | 62 | 45 | 47 | 28 | 0.135 |
| ALP (U/L) | 370 | 238 | 349 | 311 | 0.827 | ALP (U/L) | 370 | 238 | 245 | 156 | **0.03** |
| GGT (U/L) | 725 | 545 | 208 | 133 | 0.098 | GGT (U/L) | 725 | 545 | 294 | 333 | **0.022** |
|  | **Most Recent** | | | | |  | **Non-Portal HTN Group** | | | | |
| **Laboratory Data** | **Portal HTN (n= 33)** | | **No Portal HTN (n =20)** | |  | **Laboratory Data** | **Diagnosis** | | **Most Recent** | |  |
|  | **Value (Mean)** | **STD** | **Value (Mean)** | **STD** | **P-value** |  | **Value (Mean)** | **STD** | **Value (Mean)** | **STD** | **P-value** |
| ACE (nmol/mL/min) | 108 | 27 | 29 | 22 | **0.009** | ACE (nmol/mL/min) | 49 | 55 | 29 | 22 | 0.571 |
| Platelets (10^9^/L) | 137 | 83 | 214 | 108 | **0.018** | Platelets (10^9^/L) | 262 | 90 | 214 | 108 | 0.226 |
| Total Bilirubin (mg/dL) | 2.1 | 2.9 | 0.9 | 1.5 | 0.116 | Total Bilirubin (mg/dL) | 1 | 1 | 1 | 2 | 0.851 |
| Albumin (g/dL) | 3.4 | 0.7 | 4 | 0.8 | **0.008** | Albumin (g/dL) | 3.8 | 1 | 4 | 1 | 0.474 |
| AST (U/L) | 54 | 33 | 36 | 25 | 0.067 | AST (U/L) | 75 | 75 | 36 | 25 | 0.064 |
| ALT (U/L) | 47 | 28 | 39 | 26 | 0.349 | ALT (U/L) | 92 | 97 | 39 | 26 | **0.046** |
| ALP (U/L) | 245 | 156 | 155 | 121 | **0.049** | ALP (U/L) | 349 | 311 | 155 | 121 | **0.031** |
| GGT (U/L) | 294 | 333 | 441 | 126 | 0.469 | GGT (U/L) | 208 | 133 | 441 | 126 | 0.066 |

**Supplementary 2**. ***Comparison of the laboratory data drawn at time of diagnosis prior to liver biopsy and most recent laboratory data between the pHTN+ (n=33) and pHTN- (n=20) groups.*** Supplementary 2 also compares labs at time of diagnosis and most recent for the pHTN+ group (n=33) and for the pHTN- group alone. Most recent labs are drawn on average 4-6 years after initial laboratory data. Data show laboratory data for ACE level, platelets, total bilirubin, albumin, aspartate aminotransferase (AST), alanine transaminase (ALT), alkaline phosphatase (ALP), and gamma-glutamyl transpeptidase (GGT). P-value comparisons are between the pHTN+ and pHTN- groups and between labs at diagnosis and most recent within each group (T-test, p<0.05). Significant values are bolded and denoted with a *
